# Dose Reduction and Image Quality Optimization of Pediatric Chest Radiography Using a Tungsten Filter

**DOI:** 10.3390/bioengineering9100583

**Published:** 2022-10-20

**Authors:** Eunhye Kim, Kenzo Muroi, Takahisa Koike, Jungmin Kim

**Affiliations:** 1 Department of Health and Safety Convergence Science, Korea University, Seoul 02841, Korea; 2 Department of Therapeutic Radiology, Faculty of Health Care, Juntendo University, Tokyo 113-8421, Japan; 3 Department of Health Sciences, Kyorin University Graduate School, Tokyo 181-8612, Japan

**Keywords:** dose reduction, pediatric chest radiography, tungsten filter, image quality optimization

## Abstract

The use of diagnostic radiology in pediatric patients has increased, and various positive effects have been reported, including methods to reduce radiation doses in children. Research has been conducted to preserve image quality while reducing exposure and doses in pediatric patients. This study aimed to measure four different filters to identify an optimized filter for pediatric patients. The experiment was conducted using four types of filters, including aluminum, copper, molybdenum, and tungsten. The optimal filter thickness was verified using a histogram to visually evaluate the spectrum by filter thickness, effective dose on a pediatric phantom, entrance skin dose, organ absorbed dose using the PC-based Monte Carlo (PCXMC) program version 2.0 simulation, figure of merit (FOM), and image quality. As a result of measuring the spectrum according to the tube voltage and the four types of filters, dose reduction and contrast improvement effects were obtained with a 0.05 mm tungsten filter. Additionally, effective entrance skin and organ absorbed dose decreased with the said filter. The aluminum, copper, and molybdenum filters demonstrated that the effective dose scarcely decreased even when the thickness was increased; meanwhile, the effective dose decreased when the tungsten filter was 0.05 mm. The FOM with a 0.05 mm tungsten increased by 91% in the lung field and 39% in the mediastinal field. The entrance skin and organ absorbed dose in pediatric patients can be reduced by removing low-energy photons that fail in image formation using a tungsten filter.

## 1. Introduction

Owing to the intensive development of technology that uses ionizing radiation, the role of diagnostic radiology in the diagnosis and treatment of human diseases, including those in neonates and children, is expanding. According to the World Health Organization, computer and digital imaging can replace traditional film-based radiography to simultaneously send images to computers, and CT imaging is used as a beneficial tool for diagnosing diseases and injuries by increasing accuracy compared to regular radiography [1]. Fluoroscopically guided interventions can also replace surgery for diseases that occur frequently in children, thereby reducing risks [1,2]. These advances in radiation technology can significantly help in the treatment of pediatric patients; however, they can also lead to unnecessary exposure for such patients. The repeated low-energy radiation or high radiation exposure from CT and fluoroscopy during diagnostic testing is sufficient to produce deterministic effects [1,3]. Moreover, as radiation may have long-term effects, the potential and critical risks should be considered when exposing children to radiation [1,3,4].

The X-ray spectrum used in diagnostics has a wide range of photon energies, although not all of which contribute to image formation. Low energy is mostly absorbed by patients and does not contribute to image formation, while high energy causes an increase in scattering lines, which lowers the contrast [4,5]. The effects of low-energy radiation doses on patients have been evaluated by survivors of the Japanese atomic bomb and the United Nations Scientific Committee on the Effects of Atomic Radiation, where various diseases, including cancer, can be affected by radiation doses, and this risk is greater with younger age [6,7,8]. Additionally, the National Research Council US Committee on the Biological Effects of Ionizing Radiation’s BEIR V Health Effects of Exposure to Low Levels and the BEIR VII Health Risks from Exposure to Low Radiation report indicate that dose optimization for radiographic images in pediatric patients should be considered because the younger the patient is, the higher the risk of cancer [9,10].

The most important way to optimize radiation protection is to maintain doses “as low as reasonably achievable”. Various methods can be applied to reduce the radiation dose without loss of image formation, which indicates that appropriate diagnostic data images are obtained using optimal doses when using ionizing radiation [1]. The radiation dose and image quality should be optimized to balance the patient dose with the radiographic image quality, which is determined by tube voltage and additional filtration [5,11,12,13]. Although not all photons contribute to image formation, most low-energy photons, which are absorbed by the patient and do not contribute to the formation of images, have been studied for selective removal of high-energy photons [14,15].

Aluminum (Al) and copper (Cu) filters are commonly used to reduce the exposure in the continuous spectrum area of diagnostic X-rays. In this case, the overall X-ray spectral shape is essentially unchanged; therefore, a heavy element filter is used to actively utilize the K absorption edge [5,16,17]. However, these heavy element filters can only be effective if the patient’s thickness is limited to a certain range or if they are used for special medical applications. When using a bandwidth filter (K-edge filter), the appropriate tube voltage range is 70–80 kVp, which has been proven to be better for pediatric imaging, in which the subject is thin [18,19,20,21,22,23]. This study evaluated the radiation dose in patients with changes in image quality when pediatric chest radiography was performed using various filters. A suitable tube voltage was identified, and its usefulness was investigated.

## 2. Materials and Methods

The usefulness of four types of filters (Al, Cu, molybdenum [Mo], and tungsten [W]) was evaluated. The radiation generator used was a KXO-50R inverter type (Toshiba, Tokyo, Japan), the X-ray tube used was DRX 3724HD, and the image receptor used was a BaXCI (Eu) Imaging Plate (KONICA, Tokyo, Japan). The intrinsic filtration was 1.1 mm Al at 75 kVp, and the entrance dose on the image plate was kept constant using an automatic exposure control (AEC) [24].

The pediatric thorax was simulated by the phantom PBU-0 (Kyoto Kagaku Company, Kyoto, Japan) with a size of 16.0 × 17.6 × 14.5 cm^3^. This thorax diameter is representative for an age of approximately 4–5 years. This phantom is composed with urethane-based resin (density: 1.06) for soft tissue part, epoxy resin (density: 1.31) for synthetic bone part, and epoxy resin (density: 1.11) for skull. It also represents 5 bony structures (spine, clavicles, scapulae, ribs, and sternum) and 3 internal organs (lung with pulmonary vessels, trachea up to primary bronchi, and heart) as anatomically realistic shape. As for the filter material, the effective doses and image quality were evaluated at tube voltages of 60, 80, 100, and 120 kV, while changing the thickness of the filter using Al (1–12 mm), Cu (0.05–0.3 mm), Mo (0.05–0.3 mm), and W (0.05–0.3 mm). Each additional filter was added to 1 mm Al, which is an intrinsic filtration, and spectrum measurement was performed at an effective energy of 70 keV. According to previous studies, the filter thickness of the hardness corresponding to 0.2 mm Cu at an effective energy under different conditions was different depending on the conditions of the filter (Table 1).

First, the spectra at 60, 80, 100, and 120 kVp were measured using a Ramtec 413 type X-ray spectrum analyzer (Toyo Medic, Tokyo, Japan), while an additional filter was attached to determine the spectrum by the thickness of the filter. For the skin dose, the source-to-image distance was set to 150 cm, and the source-to-object distance was set to 138 cm to measure the effective dose on the pediatric phantom according to the tube voltage change and filter type. The entrance dose was measured using an EY-1002D fluorescence meter (Torex Semiconductor, Tokyo, Japan). A PC-based Monte Carlo program (PCXMC) simulation was used to calculate the effective dose by applying weighting factors; it also could apply the different types of the filters and phantom ages to calculate the organ-absorbed dose during pediatric chest scans [27,28,29]. Effective dose is calculated for the whole body; therefore, with the equivalent doses to all organs, it would account for the sensitivity of the organ to radiation [27,28,29].

Additionally, to evaluate the performance, the figure of merit (FOM) formula was applied by dividing the square of the signal-to-noise ratio (SNR) by the entrance dose [30,31]. An ROI (1 cm diameter) was placed on the lungs, heart, ribs, and vertebrae, and noise was measured through the standard deviation of pixel values from the ROI [32]. Finally, to conduct visual evaluation of image quality, image contrast was judged by using histograms, which were intended to demonstrate the clinical advantages of the optimal tube voltage and filter combination.

## 3. Results

**Figure 1:**
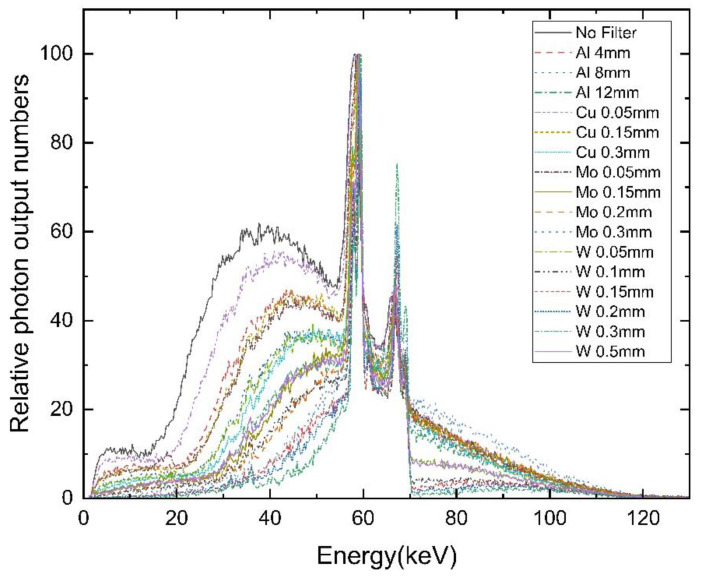
X-ray spectrum from Al, Cu, Mo, W, and no filter at 100 kV.

The spectral measurement results according to tube voltage and filter type are presented in Figure 1. As a result of the evaluation of the X-ray spectrum using an Al filter, the low-energy photons of the spectrum remained high, and when Cu and Mo filters were used, the number of low-energy photons gradually decreased as the thickness increased. In this case, the reduction in the low-energy part of ≤ 40 keV is increased with usage of a W filter. For reducing energy, the Al filter may not be practical because it displays the same photons reduction effect as the 0.05 mm W filter when it is 8 mm thick. Therefore, based on the spectral form by the W filter, the organ absorbed dose can be reduced due to the removal of the low energy dose.

**Figure 2:**
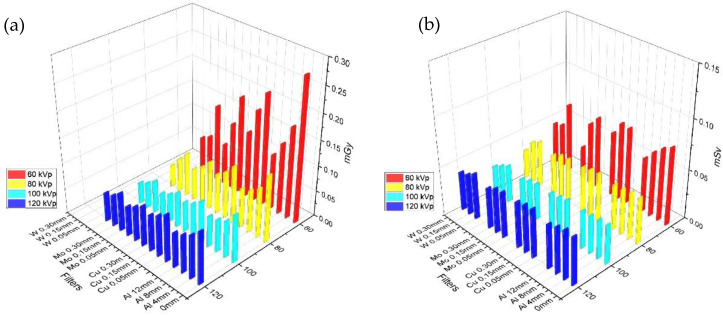
(**a**) Entrance skin dose result using various filters (Al, Cu, Mo, W, and no filter). (**b**) Effective dose result using various filters (Al, Cu, Mo, W, and no filter) at 60, 80, 100, and 120 kVp.

The entrance dose on the X-ray receptor sensor was maintained using the AEC. The experimental results according to the tube voltage and filter thickness are presented in Figure 2a. The dose value of the vertical axis is not an absolute value optimized for pediatric chest radiography but a relative value for comparing entrance skin doses by tube voltage and filters. As the thicknesses of the Al, Cu, Mo, and W filters increased, the entrance skin dose decreased gradually at 100 kVp. Thus, as the thickness of the filter increased, the overall entrance skin dose decreased; however, as the tube voltage increased, the dose reduction effect decreased, and when it reached 120 kVp, the entrance skin dose increased. When compared to the tube voltage of 100 kVp, the entrance skin dose was 1.0, in the absence of a filter. The entrance skin dose was reduced as follows: Al 4 mm, 0.77; Cu 0.05 mm, 0.85; Mo 0.05 mm, 0.73, and W 0.05 mm, 0.68, indicating that the dose reduction effect of the W filter was the greatest.

**Figure 3:**
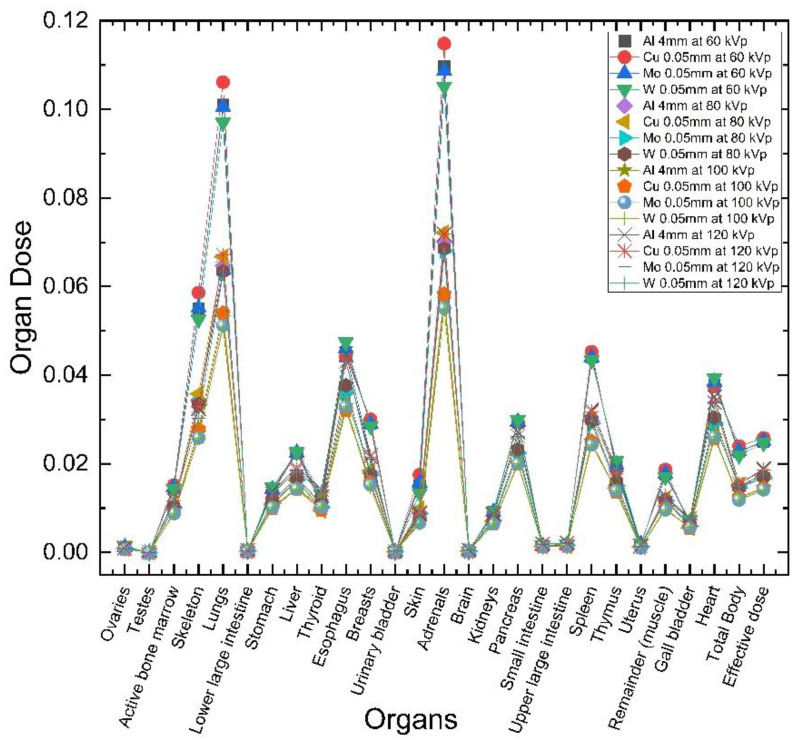
Organ absorbed dose result using various filters (Al, Cu, Mo, W, and no filter) at 60, 80, 100, and 120 kVp.

For effective and organ absorbed doses, Monte Carlo simulations were performed using the PCXMC software based on the entrance skin dose [27,28,29]. The statistical uncertainties of the resulting doses from PCXMC were when the simulation was set to 200,000, and the relative errors were below 5% for organs in the X-ray field [32]. Organ absorbed dose values, such as those in the bones, thyroid, lungs, bone marrow, breasts, testis, and ovaries were obtained using tube voltage, four filters, and no filters in pediatric chest imaging (Figure 2b and Figure 3). For this, the organ absorbed dose calculation by using the PCXMC was applied with the tissue weighting factor presented by ICRP publication 103 [33]. Among children, especially newborns, the risk of increasing organ absorbed doses is high because they are small in body size and are often subject to total body irradiation even when only the chest area requires imaging. Even when the X-ray fields were limited to the chest alone, the lungs and bones had the highest organ absorbed dose, followed by the breast bone marrow and thyroid gland. Depending on the filter type, the effective dose was the highest when the 4 mm Al filter was used, followed by 0.05 mm Cu, 0.05 mm Mo, and 0.05 mm W. Therefore, the W filter had the greatest organ absorbed dose reduction, especially at 60 kVp, compared with the other filter types, and no significant difference was observed at > 80 kVp. The Al, Cu, and Mo filters indistinctly decreased the effective dose even when the thickness was increased. Meanwhile, when the W filter was 0.05 mm, the effective dose was 0.023, indicating a decrease of 9%; when the W filter was 0.01 mm, the effective dose was 0.021, indicating a decrease of 22%.

**Figure 4:**
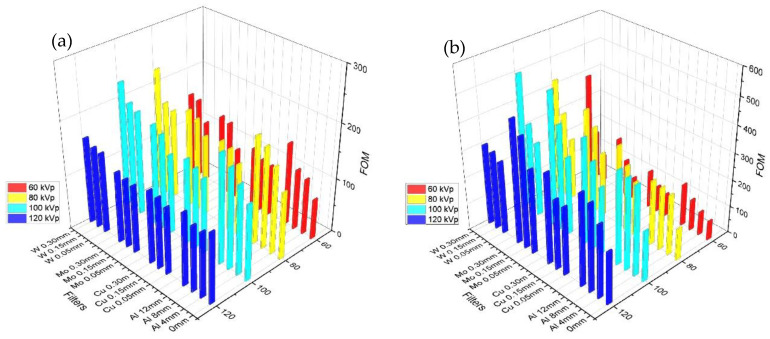
(**a**) FOM of lung field result using various filters (Al, Cu, Mo, W, and no filter), (**b**) FOM of heart field result using various filters (Al, Cu, Mo, W, and no filter) at 60, 80, 100, and 120 kVp.

The FOM is used to evaluate the performance by dividing the square of the signal-to-noise ratio (SNR) by the entrance dose [30,34,35]. The performance displays the FOM of the lung and mediastinal areas separately (Figure 4a,b, respectively). With no filter, the FOM value of the lung field increased according to the voltage. In all the types of filters, if the tube voltage increases to 100 kVp, the FOM value also increases; thus, the FOM is highest at 100 kVp; however, if the tube voltage increases more, the FOM value decreases. Although the increase in the Al and Cu filters was not significant, depending on the type of filter, the FOM significantly increased when using the Mo and W filters as the thickness of the filter increases. Hence, the Mo and W filters demonstrated a greater dose reduction effect than the Al and Cu filters. The FOM of the 0.05 mm W filter increased by 91% in the lung field and 39% in the mediastinal field at 100 kVp.

**Figure 5:**
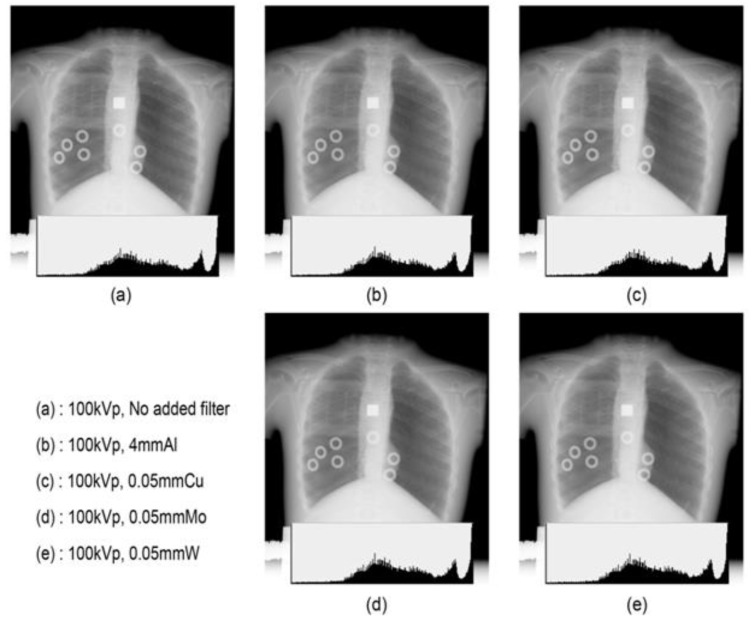
Image quality and histogram of various filters (Al, Cu, Mo, W, and no filter).

During adult chest radiography, contrast is often lowered by increasing the tube voltage to widen the latitude. However, in pediatric cases, the degree of latitude needs not be increased by increasing the tube voltage or lowering the contrast because the subject contrast of the thoracic organs is small, and the air content of the lungs is low. Phantom images were compared with a tube voltage fixed at 100 kVp and using different filter types; Figure 5a presents an image without a filter, while the remaining images in the figure are pediatric chest images with the Al, Cu, Mo, and W filters with a similar transmission energy. However, distinguishing the difference in contrast or noise of all images was not possible, and the difference in image quality could not be identified, even in the histogram. Digital chest radiographs demonstrate that the change in image quality according to the tube voltage or type of filter is insignificant, and the optimal condition can be determined by considering the entrance dose, effective dose, etc.

## 4. Discussion

Generally, the use of additional filters is effective in improving the X-ray effective energy by reducing the low-energy range; however, the use of additional thin filters is inevitable because of the reduction in image contrast due to X-ray quality enhancement [36]. In this study, we investigated the relationship between patient dose and image quality using appropriate filters and kVp values in pediatric chest radiography. For the Al, Cu, and Mo filters, the number of X-ray photons decreased in the low-energy region, whereas no changes in the high-energy region were observed. However, the W filter has been demonstrated to remove not only low-energy photons but also high-energy photons that fail in image formation by forming a bandwidth shape around a specific energy. The entrance skin dose (ESD) decreased as the kVp and filter thickness increased. The ESD of the 4 mm Al filter was the highest, followed by the 0.05mm Cu, 0.05 mm Mo, and 0.05 mm W filters.

The Al, Cu, and Mo filters demonstrated that the effective dose was insignificantly decreased even when the thickness was increased. The effective dose was 0.023 when the W filter was 0.05 mm, the effective dose was decreased by 9% to 0.021 when the filter was 0.1 mm, and the effective dose was decreased by 22% to 0.018 when the filter was 0.3 mm. Through PCXMC simulation, it was found that the lungs and bones received the highest radiation exposure among the pediatric organs. In addition, it was found that the organ absorbed dose reduction was the highest with the W filter among the filter types.

The FOM did not change significantly when using the Al and Cu filters but increased significantly when using the Mo and W filters, although the difference between the images was insignificant. The change in the mediastinal FOM was similar to that of the pulmonary FOM; however, using the 0.05 mm W filter increased the pulmonary FOM by 91% and that of the mediastinal membrane by 39%. The FOM increased with the addition of the filter, which can be attributed to the lower overall radiation dose exposed to patients [37].

The optimized condition for digital pediatric chest radiography was a 0.05 mm W filter, which reduced ESD by 32% and effective dose by 2% compared with applying no filter. This optimized condition reduced unnecessary doses in pediatric chest radiography without loss of image quality. In previous studies using W filters, which increased the overall image quality, the entrance dose decreased from 22% to 15%, and the overall image quality was more uniform [17]. Other studies have also reported that the addition of a 0.1mm W filter reduced the total absorbed dose by 0.73 times and did not reduce the overall image quality [15]. Additionally, a study has reported that the W filter, which is the bandwidth filter, reduced the radiation exposure of patients by >50% compared to the Al filter without decreasing the image quality [5,38,39].

When using a filter, the tube voltage or tube current can be increased to adjust the number of photons irradiated to the patient; however, care should be taken regarding increasing the exposure time as it has a decisive effect on image quality by increasing the degree of dissonance due to movement. Additionally, the exposure time should be short in newborns or children who frequently move. According to the American College of Radiology standards, adult radiograph exposure time should not be >40 ms, and the degree of disconnection due to movement may be evident if the exposure time is 20 ms during pediatric radiograph; nevertheless, the quality of movement should be significantly reduced to <10 ms.

## 5. Conclusions

Radiation exposure can have side effects, including cancer, and the risk decreases with age. Thus, pediatric patients are also vulnerable to the risk. To reduce the effective and organ absorbed dose exposed to pediatric patients while maintaining image quality, experiments on patient dose and image quality changes were conducted using four filters: Al, Cu, Mo, and W. The results indicate that W, a heavy element filter, could increase image quality while reducing the effective and organ absorbed doses in a patient. Hence, the use of a W filter is effective in improving the image quality of X-ray images and reducing the organ absorbed dose of patients.

## Figures and Tables

**Table 1:** Filter thickness being hardness equivalent to 0.2 mm copper depends on the filter material [18,25,26].

Filter Material	K-Edge (keV)	Equivalent Thickness (mm) (Effective Energy (keV))			
		This Work (70 keV)	Nagel, H (30 keV)	Jennings (65 keV)	Koedooder/Venema (70 keV)
Aluminium (Al)	0.56	6.1	7.1	7.2	5.77
Coppper (Cu)	8.98	0.2	0.2	0.2	0.2
Molybdenum (Mo)	20	0.076			0.076
Tungsten (W)	69.5	0.045	0.046		0.046

## Data Availability

Not applicable.

## References

[1] World Health Organization (2016). Communicating Radiation Risks in Paediatric Imaging: Information to Support Healthcare Discussions about Benefit and Risk.

[2] Tapiovaara M.J., Sandborg M., Dance D.R. (1999). A search for improved technique factors in paediatric fluoroscopy. Phys. Med. Biol..

[3] Kim E., Boyd B. (2022). Diagnostic Imaging of Pregnant Women and Fetuses: Literature Review. Bioengineering.

[4] Carlsson G.A., Chan H.P. (1999). Commentary: Progress in optimization of patient dose and image quality in X-ray diagnostics. Phys. Med. Biol..

[5] Villagran J.E., Hobbs B.B., Taylor K.W. (1978). Reduction of patient exposure by use of heavy elements as radiation filters in diagnostic radiology. Radiology.

[6] United Nations Scientific Committee on the Effects of Atomic Radiation (UNSCEAR) (2008). Annex A. Epidemiological Studies of Radiation and Cancer. 2008a UNSCEAR 2006 Report.

[7] Little M.P. (2009). Cancer and non-cancer effects in Japanese atomic bomb survivors. J. Radiol. Prot..

[8] Little M.P. (2001). Comparison of the risks of cancer incidence and mortality following radiation therapy for benign and malignant disease with the cancer risks observed in the Japanese A-bomb survivors. Int. J. Radiat. Biol..

[9] The National Academics (1990). Health Effects of Exposure to Low Levels of Ionizing Radiation: BEIR V Board on Radiation Effects Research.

[10] The National Academics (2006). Health Risks from Exposure to Low Levels of Ionizing Radiation: BEIR VII Phase 2. Board on Radiation Effects Research.

[11] Hamer O.W., Sirlin C.B., Strotzer M., Borisch I., Zorger N., Feuerbach S., Völk M. (2005). Chest radiography with a flat-panel detector: Image quality with dose reduction after copper filtration. Radiology.

[12] Shrimpton P.C., Jones D.G., Wall B.F. (1988). The influence of tube filtration and potential on patient dose during X-ray examinations. Phys. Med. Biol..

[13] Hata A., Yamada Y., Tanaka R., Nishino M., Hida K., Hino T., Ueyama M., Yanagawa M., Kamitani T., Kurosaki A. (2021). Dynamic Chest X-ray Using a Flat-Panel Detector System: Technique and Applications. Korean J. Radiol..

[14] Berfer M.J., Motz J.W. (2004). X-rays from thick tungsten targets irradiated by 500–50 keV electrons. Sci. Direct.

[15] Yamaguchi C., Yamamoto T., Terada H., Akisada M. (1983). Effect of tungsten absorption edge filter on diagnostic X-ray spectra, image quality and absorbed dose to the patient. Phys. Med. Biol..

[16] Kim Y. (2000). A study on the reduction of exposure dose and contrast improvement by use of heavy elements filter. J. Orient. Technol..

[17] Chu J.C., Galvin J.M., Lockett P., Bloch P. (1981). Use of a tungsten filter to improve beam uniformity. Med. Phys..

[18] Nagel H.D. (1989). Comparison of performance characteristics of conventional and K-edge filters in general diagnostic radiology. Phys. Med. Biol..

[19] Kim G.S., Kim S.C. (2015). Comparison of image quality and effective dose by additional filtration on digital chest tomosynthesis. J. Radiol. Sci. Technol..

[20] Atkins H.L., Fairchild R.G., Robertson J.S., Greenberg D. (1975). Effect of absorption edge filters on diagnostic X-ray spectra. Radiol..

[21] McParland B.J., Boyd M.M. (2001). X-ray image intensifier performance and patient doses for combinations of supplemental beam filters and vascular contrast agents. Phys. Med. Biol..

[22] Nagel H.D. (1988). Limitation in the determination of total filtration of X-ray tube assemblies. Phys. Med. Biol..

[23] Toroi P., Zanca F., Young K.C., Ongeval C., Marchal G., Bosmans G.H. (2007). Experimental investigation on the choice of the tungsten/rhodium anode/filter combination for an amorphous selenium-based digital mammography system. Eur. Radiol..

[24] Onnasch D.G.W., Schemm A., Kramer H. (2004). Optimization of radiographic parameters for paediatric cardiac angiography. Br. J. Radiol..

[25] Jennings R.J. (1988). A method for comparing beam-hardening filter materials for diagnostic radiology. Med. Phys..

[26] Koedooder K., Venema H.W. (1986). Filter materials for dose reduction in screen-film radiography. Phys. Med. Biol..

[27] Yakoumakis E., Dimitriadis A., Makri T., Karlatira M., Karavasilis E., Gialousis G. (2013). Verification of radiation dose calculations during pediatric cystourethrography examinations using MCNP5 and PCXMC 2.0 Monte Carlo codes. Radiat. Prot. Dosim..

[28] Brosi P., Stuessi A., Verdun F.R., Vock P., Wolf R. (2011). Copper filtration in pediatric digital X-ray imaging: Its impact on image quality and dose. Radiol. Phys. Technol..

[29] Tapiovaara M., Siiskonen T. (2008). PCXMC, a Monte Carlo program for calculating patient doses in medical X-ray examinations. Radiat. Prot. Dosim..

[30] Smans K., Struelens L., Smet M., Bosmans H., Vanhavere H.F. (2010). Cu filtration for dose reduction in neonatal chest imaging. Radiat. Prot. Dosim..

[31] Iramina H., Hamaguchi T., Nakamura M., Minzowaki T., Kanno I. (2018). Metal artifact reduction by filter-based dual-energy cone-beam computed tomography on a bench-top micro-CBCT system: Concept and demonstration. J. Radiat. Res..

[32] Borrego D., Lowe E.M., Kitahara C.M., Lee C. (2018). Assessment of PCXMC for patients with different body size in chest and abdominal X-ray examinations: A Monte Carlo simulation study. Phys. Med. Biol..

[33] International Commission on Radiological Protection (2007). The 2007 Recommendations of the International Commission on Radiological Protection.

[34] Aichinger H., Dierker J., Joite-Barfuß S., Säbel M. (2004). Radiation Exposure and Image Quality in X-ray Diagnostic Radiology.

[35] Dobbins J.T., Samei E., Chotas H.J., Warp R.J., Baydush A.H., Floyd C.E., Ravin C.E. (2003). Chest radiography: Optimization of X-ray spectrum for cesium iodide-amorphous silicon flat-panel detector. Radiology.

[36] Kim S.H., Choi J.H. (2015). Analysis of effectiveness of spectrum of energy and image quality evaluation by aluminium filter in the added compound filtration. J. Radiol. Sci. Technol..

[37] Doyle P., Martin C.J., Gentle D. (2006). Application of contrast-to-noise ratio in optimizing beam quality for digital chest radiography: Comparison of experimental measurements and theoretical simulations. Phys. Med. Biol..

[38] Regano L.J., Sutton R.A. (1992). Radiation dose reduction in diagnostic X-ray procedures. Phys. Med. Biol..

[39] Park H.S., Kim Y.S., Kim S.T., Park O.S., Jeon C.W., Kim H.J. (2011). Survey of technical parameters for pediatric chest X-ray imaging by using effective DQE and dose. Korean Soc. Med. Phys..

